# Self-control in Online Discussions: Disinhibited Online Behavior as a Failure to Recognize Social Cues

**DOI:** 10.3389/fpsyg.2017.02372

**Published:** 2018-01-11

**Authors:** Birgit J. Voggeser, Ranjit K. Singh, Anja S. Göritz

**Affiliations:** Department of Psychology, Albert-Ludwigs-Universität Freiburg, Freiburg im Breisgau, Germany

**Keywords:** online disinhibition, self-control, ego-depletion, social cues, flaming, trolling

## Abstract

In an online experiment we examined the role of self-control in recognizing social cues in the context of disinhibited online behavior (e.g., flaming and trolling). We temporarily lowered participants' self-control capacity with an ego depletion paradigm (i.e., color Stroop task). Next, we measured participants' sensitivity to social cues with an emotional Stroop task containing neutral, negative, and taboo words. Sensitivity to social cues is represented by the increase in reaction time to negative and especially taboo words compared to neutral words. As expected, undepleted participants were slower to process the color of negative and taboo words. By contrast, depleted participants (i.e., those with lowered self-control capacity) did not react differently to taboo or negative words than they did to neutral words. The experiment illustrates that self-control failure may manifest itself in a failure to recognize social cues. The finding underlines the importance of self-control in understanding disinhibited online behavior: Many instances of disinhibited online behavior may occur not because people are unable to control themselves, but because they do not realize that a situation calls for self-control in the first place.

## Introduction

The Internet has revolutionized the way humans exchange ideas, learn from one another, and coordinate collective action. It facilitates fast and effortless communication to small and large audiences. These new forms of social interaction have enriched both personal lives and societies. However, in addition to amplifying and spreading constructive discourse, the Internet can also amplify and spread instances of uncivil, inappropriate, or disinhibited communication (e.g., Joinson, 2003, 2007; Suler, 2004), which are referred to as *toxic online disinhibition* (Suler, 2004; Lapidot-Lefler and Barak, 2012).

In this paper, we look at toxic online disinhibition from the new perspective of self-control. Viewing toxic online disinhibition as a form of self-control failure offers insights and informs future research. The empirical portion of this paper presents an online experiment in which we isolated an elusive type of self-control failure that appears to play a crucial role in many instances of toxic online disinhibition: the failure to recognize relevant social cues. Before presenting our study, we provide an overview of the forms of toxic online disinhibition and summarize existing theories.

*Flaming*, which is an aggressive verbal outburst by one or more participants in online-discussions, is the most commonly observed manifestation of toxic online disinhibition (Alonzo and Aiken, 2004; Johnson et al., 2009). Flaming occurs on impulse and appears to be a defensive reaction to a perceived insult or unacceptable opinion expressed by others. While single instances of flaming can disrupt the well-being of an online community, a larger problem arises when users influence each other's communication behavior (Papacharissi, 2004; Lapidot-Lefler and Barak, 2012; Anderson et al., 2014). One person's incivility can be sufficient to start a *flame war*, which is a major user-on-user group-conflict within a community. Users group into factions with strong opinions on polarized topics and attack the other faction(s) with violent language (Johnson et al., 2009). Another phenomenon that can arise from single instances of uncivil online communication is the “*shitstorm*,” which occurs when a large group of people voice their discontent with one entity (this entity can be anyone from a single person to any form of organization) using different social media platforms in an unrestrained manner. While the term “shitstorm” refers to any instance of verbally violent uproar, it is increasingly used in the online context most likely because the Internet intensifies visibility, frequency, and severity of “shitstorms.” Both flame wars and “shitstorms” derail societal and political discourse, hinder consensus finding and impede progress for smaller communities and entire societies.

In addition to instances of spontaneous incivility, there are prominent forms of purposeful uncivil online behavior, which are usually referred to as *trolling* (Hardaker, 2010). Trolling describes the act of intentionally derailing discourse and inciting those involved in a discussion to start flaming. Trolling disrupts meaningful discourse, undermines community functionality and cohesion and leads to flaming along with all its consequences.

Intentional incivility directed toward particular persons can manifest as cyber-bullying or cyber-stalking, which are the online-counterparts to bullying, mobbing, and stalking behaviors offline (Privitera and Campbell, 2009; Slonje et al., 2013). Often, the perpetrator also harasses the victim offline, but the Internet exacerbates the problem: bullying and stalking online are boundless because the restrictions that can be applied offline (e.g., restraining orders) are impossible to uphold online. Furthermore, the victims cannot evade or escape their perpetrators; blocked accounts can be replaced with new accounts, and new communities can be joined under a false identity. As soon as the perpetrator determines the victim's virtual whereabouts, they can resume the harassment. The easy access to a victim's social circles via social networks holds more potential for a perpetrator to ruin their victim's private and/or work life. One example is “revenge porn,” where a former romantic partner posts intimate pictures or films of the victim online as revenge for terminating the relationship (Davies, 2015). The consequences for the victims can be as severe as those that result from offline-bullying or stalking: unraveling of their work and private life often culminating in mental health issues or even suicide (Kowalski et al., 2014).

Undoubtedly, disinhibited online communication is a pressing concern for both private and public stakeholders. The question is how the Internet increases the frequency and intensity of disinhibited online behavior. In general, there are two possible and not mutually exclusive explanations: (1) The Internet amplifies the dissemination of aggressive messages that would exist without it but would not have reached a substantive audience via traditional media and (2) the Internet affects communication behavior, thus increasing the likelihood of people to communicate aggressively online.

The Internet amplifies the dissemination of aggressive messages because it facilitates the spreading of messages regardless of sender and content. Thus, people are now able to share aggressive messages with the world more than previously via traditional media. Moreover, these messages can now reach larger audiences through snowballing via online sharing. Once shared, these messages are easier to access than messages communicated using traditional media because they are usually accessible from anywhere worldwide for a long time after they have been created.

A lot of psychological research into disinhibited online communication focuses on the second possibility: the Internet affects communication behavior. Theories have emerged that focus on mechanisms or groups of mechanisms explaining how the Internet as a medium fosters aggression and incivility in the people who use it.

Most theoretical approaches explaining disinhibited online communication are based on established theories, predominantly deindividuation, media-richness, social, situational, and environmental cues, as well as identity theories (Döring, 2003; Joinson, 2007).

An often-cited theory is the Social Identity model of Deindividuation Effects (SIDE, Spears and Lea, 1992), which extends Zimbardo's (1970) deindividuation theory. SIDE consists of two components: First, the cognitive component posits that different sets of norms and behavioral goals are activated when interacting with others due to either group salience or a focus on one's individuality. Second, the strategic component posits that anonymity is used strategically to level out hierarchies and act against established norms of social conduct without reprimand (Spears and Lea, 1992; Christopherson, 2007; Joinson, 2007).

The theory of reduced social cues associates online disinhibition with a lack of social cues to control inherent in an online environment stripped of contextual information regarding the interaction partners (Kiesler et al., 1984; Döring, 2003). Without the cues to control, norms that are common in face-to-face-interactions do not manifest online, and therefore, norm adherence is reduced. This approach ties in with the broader field of media richness research.

An integrative approach proposed by Suler (2004) combines different theorized factors that may lead to online disinhibition into a single model. This model focuses on several aspects of anonymity that are common to many online interactions and their effects on an individual's behavior toward others. Suler coined the term “online disinhibition effect.”

However, Lapidot-Lefler and Barak (2012) indicate that the largely anonymous architecture of the Internet is not the only, and potentially not the largest, factor that leads to online disinhibition (OD). Many instances of OD occur in mostly non-anonymous online environments, such as Facebook and Twitter. People post inappropriate, hostile, or incriminating messages under their real names and next to pictures of themselves. Often, posts are visible to friends, family and work contacts, which can lead to direct and substantial consequences. One potential consequence that has received media attention is “Facebook-firings,” which refers to the termination of employment due to insulting comments toward an employer on social networks (Bacharach, 2011).

In addition to theories that focus on factors that affect all users equally, differential approaches attempt to identify attributes that make users particularly susceptible to OD. These approaches identify personality traits that in- or decrease the likelihood of disinhibited online communication. One of the two personality models that are predominantly used in this context is the Big 5 model of personality, which spans the five personality factors of openness, conscientiousness, extraversion, agreeableness, and neuroticism (O'Keefe et al., 2012). For example, Kokkinos et al. (2013) found that high extraversion and conscientiousness lower the likelihood that an individual engages in cyberbullying.

The other personality model is the Dark Tetrad, which is an extension of the Dark Triad. The Dark Tetrad comprises the three factors subclinical psychopathy (lack of empathy), Machiavellianism (urge to manipulate and control others), and narcissism (excessive self-love), and the added fourth factor everyday sadism (subclinical urge to hurt others or see them suffer; Mededović and Petrović, 2015). Buckels et al. (2014) found positive correlations between everyday sadism and the propensity to engage in trolling. They also report weaker effects for subclinical psychopathy and Machiavellianism. Most of these approaches examine purposeful incivility, cyberbullying, and trolling, which, while often more severe, constitute a smaller proportion of OD than spontaneous incivility (Johnson et al., 2009; Hardaker, 2010; Buckels et al., 2014).

While existing theories have proposed likely causes of OD, we feel that examining disinhibited online behavior from a more integrative perspective is worthwhile. As suggested by the word “disinhibited,” disinhibited behavior is a form of self-control failure. Vohs et al. (2008) define self-control “as the self-exerting control to override a prepotent response with the assumption that replacing one response with another is done to attain goals and conform to standards.” This definition describes the one feature that is shared by all forms of OD: They are a violation of social and/or legal norms. To illustrate why looking at OD as self-control failure is promising, we draw on the basic idea of Carver and Scheier's (2004) conception of self-control as a feedback control system: Self-control is governed by a person's goals (or norms or standards). To ensure that a goal is reached, people need to monitor their current internal and external situation. To do so, people need to watch out for cues relevant to their goal. Then, people must compare the perceived situation with the intended goal. If there is a mismatch between the current situation (“is”) and the intended goal (“should/ought”), people need to modify their behavior. Modifying behavior might entail initiating or intensifying goal-directed behaviors and reducing or stopping behaviors that are incompatible with the goal. Self-control failure occurs if any of these three components of the feedback loop fails. In the context of online communication, to communicate appropriately, people must intend to do so, realize which messages are in-/appropriate in a particular context, and then, modify their behavior accordingly.

This perspective implies three categories of causes leading to OD: (1) OD occurs if people do not intend to communicate appropriately online; (2) OD occurs when people intend to communicate appropriately and realize that they should modify their behavior but are unable to modify their behavior; and (3) finally, OD occurs when people intend to communicate appropriately and are able to modify their behavior but fail to realize that they should modify their behavior.

This three-pronged perspective on OD accounts for different mechanisms that lead to incivility online and allows for deriving tailor-made solutions that are appropriate for each mechanism. Additionally, this three-pronged perspective inspires new approaches for research and practical applications.

If people do not intend to communicate appropriately online, they make no effort to inhibit inappropriate communication behavior. In certain cases, these people may even invest effort into communicating in a toxic manner. Manifestations of intentional incivility include trolling and cyberbullying (e.g., Hardaker, 2010; Slonje et al., 2013). Studies investigating this phenomenon typically focus on two causes: a lack of norms (e.g., Kiesler et al., 1984) and a (perceived) lack of consequences (e.g., Suler, 2004).

Most people intend to communicate appropriately simply because they share underlying social norms, such as the belief that it is wrong to hurt other people. The Internet has been argued to be a norm-free space without ground rules (Kiesler et al., 1984). However, studies have cast doubt on this hypothesis and have demonstrated that even physical norms, such as personal space and gaze direction, are transferred to the virtual environment (Yee et al., 2007). Communication norms also manifest, such as the rules for good behavior online that are referred to as netiquette (WebWise Team, 2012). Park et al. (2014) show that adolescent online users who internalized netiquette-rules are less likely to engage in cyberbullying.

However, not all online users hold pro-social norms. For example, users with anti-social personality traits do not hold these norms. As mentioned above, Buckels et al. (2014) showed that people with stronger sadistic personality traits are more likely to troll than people with weaker sadistic tendencies. It can be argued that these people cause conflict intentionally rather than by a temporary, unintended lapse in self-control. In this case, the norms that guide their behavior vary from those held by the general online population. However, while moderate expressions of antisocial personality traits are widespread (Buckels et al., 2013, 2014), people with strong antisocial tendencies and, therefore, strongly divergent norms are a minority (Torgersen et al., 2001).

The suspension or absence of communication norms may also occur when users encounter diverging opinions they deem immoral or unacceptable. The perceived wrongness may elicit an emotional hot state, often leading users to break the rules of conduct to assert their own views as the rightful ones. Since they consider themselves to be correct and the holders of the diverging opinions to be incorrect on a moral level, the norms of pro-sociality and good conduct are deliberately disregarded. Certain users actively seek diverging opinions and intentionally engage in heated debates with others. The various explanations for these behaviors include an effort to “fix” the others' wrong opinions and the pleasure experienced during heated debates. The act of deliberately seeking out content that leads to this emotional hot state is referred to as “hate reading” (Baker, 2012).

Furthermore, different online contexts, such as different social networks, have their own set of norms that deviate from the mainstream. For example, rant-sites are online communities with the explicit goal of ranting—venting frustration—about specific topics. Here, the norms demand OD (Martin et al., 2013).

However, even if someone does hold a norm that would justify communicating impolitely, they might still intend to comply with societal norms of politeness due to fear of negative consequences. In this context, anonymity is often cited as a cause for OD (e.g., Suler, 2004; Christopherson, 2007). Anonymity allows users to share extreme views or verbally attack other users without consequences in their everyday lives. However, as online communication increasingly ceases to be anonymous, such as communicating via Facebook, a reduction in OD would be expected. However, studies have shown that the absence of anonymity does not prevent OD (e.g., Lapidot-Lefler and Barak, 2012). This finding could be explained by a mismatch between actual accountability and perceived accountability. Alternatively, people do not consider all outcomes when engaging in OD. Altogether, anonymity does not appear to be the main culprit, and making users identifiable does not appear to be effective enough to curb OD.

OD can occur when people attempt to control their communication behavior but fail to succeed. This is perhaps the most relatable type of self-control failure in communication: unsuccessfully attempting to withhold a toxic response to a post or joining an unconstructive online argument even though one knows it is pointless.

Many factors online increase the likelihood of self-control failure despite self-control intentions. The Internet, as a medium, may challenge people's self-control capacity due to technical problems, long loading times, or poor design. The likely resulting “Internet rage” (Bratskeir, 2015) interferes with controlling behavior online. Similarly, many usage situations on the Internet may be challenging for self-control, such as using the Internet in a distracting environment during a commute. In other usage situations, users may be less able to control themselves because they are exhausted after a long day at work or school (Banks and Dinges, 2007).

OD can occur when people intend to act appropriately and are able to modify their behavior but fail to realize that they act inappropriately and should modify their behavior. Perceiving and processing relevant internal or external cues to control is the first step of successful self-control (Carver and Scheier, 1998). If cues are not processed appropriately, no further steps of self-control can ensue. In our research, we focus on this third type of OD because it may explain the widespread nature of OD: Many usage situations on the Internet are not conducive to attentively monitoring one's behavior. When a person is sitting at home and relaxing after a long day at work, they do not usually pay attention to their behavior. Other usage contexts are fraught with distraction, such as using the Internet on a mobile device during a commute, or simultaneously to other activities, such as watching TV (Székely, 2015).

Moreover, the Internet as a medium may make lapses in monitoring more likely, because users are not reminded to monitor their behavior as frequently and saliently as offline. Social behavior is directed not only by conscious intent and control but also by a myriad of subtle cues to control (e.g., Holler and Beattie, 2003). This argument ties into media richness research, which looks at how much information a medium transports and the vividness of the information (Suh, 1999). Online communication often lacks the subtle social cues to control that are available in face-to-face communication. One example is eye contact. Direct eye contact, even with one's own reflection (Carver and Scheier, 1981) or a picture of an eye (Oda et al., 2015), increases self-awareness and the monitoring of one's behavior. Since attention directed toward one's behavior is the first step in controlling behavior, this increase in monitoring increases the likelihood of successful self-control.

Lapidot-Lefler and Barak (2012) demonstrate this effect in computer-mediated communication. The authors deconstructed anonymity, which hides several cues at once, by testing which of the cues that are lost due to anonymity have the strongest impact. By removing eye contact only while still providing participants with their counterparts' name, sight of them and all contextual information included in seeing them, OD increased. Due to the randomized design, this increase cannot be explained by differing norms, the intent to communicate appropriately or differences in the participants' self-control capacity.

The Internet, however, is not devoid of social cues. Different compensation strategies have emerged by which users attempt to compensate for the lack of contextual and social cues. Emotes and smileys are used to convey emotion via stylized facial expressions; text-formatting, such as bold print and italics, is used to convey emphasis; and writing in capital letters is used to emulate shouting. While these artificial cues have an effect (Derks et al., 2007), the lack of immediate and often involuntary cues that are found in synchronous and certain forms of mediated communication, such as telephone or face-to-face communication, cannot be fully compensated for.

This study focuses on the type of self-control failure: People who do not detect cues to self-control and, thus, do not realize that their communication behavior is (or about to be) inappropriate. OD is likely a multi-causal phenomenon, and the aspects that can be isolated theoretically are interdependent in practice. However, unconscious self-control failure complements the existing perspectives on OD because this failure can be used to explain OD in well-adjusted users with a normal self-control capacity. Since the failure occurs upstream in the self-control process, all elements of the self-control loop do not come into play. The strength of an individual's aims and norms and their impulse control are irrelevant if the individual does not realize that their communication is amiss or awry. Furthermore, since users may not, or not fully, realize that the communication was inappropriate even after the communication occurred, the motivation to change their behavior in the future is lacking. In our study, we aim to induce and isolate unconscious self-control failure to demonstrate that this type of failure is distinct from a failure of impulse control.

Our study has two goals: (1) experimentally demonstrate that a lapse in self-control reduces sensitivity to social cues to control and (2) show that these experiments are feasible in an online setting using only native web technologies without plug-ins. Therefore, our study relies on two components: manipulating the participants' state self-control capacity and detecting that the participants fail to recognize social cues to control rather than fail to control their reaction to recognized cues.

Manipulating the participants' state self-control capacity is necessary for revealing the causal relationship we propose. Thus, we rely on the ego depletion effect. Ego depletion refers to a phenomenon in which people who have exerted self-control effort are temporarily less able to control themselves afterwards (Baumeister et al., 1998). In other words, exerting self-control reduces people's state self-control capacity for a short duration. While an initial meta-analysis by Hagger et al. (2010) found ego depletion to be a substantial and reliable effect, a subsequent meta-analysis performed by Carter et al. (2015) revealed contradictory results. Applying several meta-analytic corrections for publication bias, the authors estimated that the ego depletion effect is smaller than previously assumed. One correction even implied a null-effect. The most recent meta-analysis performed by Dang (2017) addressed some shortcomings of the second meta-analysis (Carter et al., 2015) and yielded differing results: Ego depletion, while not as strong as observed in the first meta-analysis, appears to be a real effect. The meta-analysis also confirms that the Color Stroop task is an effective method of inducing ego depletion. The specific Color Stroop task adaptation used in our study has been associated with depletion in a series of two experiments (Singh and Göritz, submitted).

Most studies using ego depletion focus on the phenomenon itself and the consequences of prior self-control exertion in different contexts (Hagger et al., 2010). However, ego depletion can also be used to gain insight into self-control dynamics in general, because it allows researchers to temporarily lower the participants' self-control capacity. Thus, ego depletion allows for experimental investigations of the causal effects of self-control capacity.

By reducing the participants' state self-regulation, we can simulate situations and conditions that occur in people's every-day lives and accordingly in their online interactions: (1) we simulate situations in which a person is engaged in online interaction while their self-regulation capacity has been depleted by previous taxing tasks or duties, such as a long day at work, partaking in mentally vexing online activities or conflict in private life; (2) we simulate situations in which people have a lowered self-control capacity due to parallel self-control demands, such as distractions due to multi-tasking, interactions with family members, or noise due to neighbors or traffic; and (3) we simulate people who have a low trait-self-regulation capacity due to being part of a demographic segment with lower self-regulation-capacities (de Ridder et al., 2012) or have a mental or physical condition that lowers self-regulation-capacity, such as chronic pain.

To determine whether people fail to process social cues to control, we employed a modified version of the emotional Stroop task. In the emotional Stroop paradigm, the participants are asked to identify the color in which several words are presented. Unlike the color Stroop paradigm, the emotional Stroop paradigm presents words that have no color meaning but differ in valence. The participants are slower to identify the color of emotional words (positive or negative valence) than the color of neutral words (Eilola et al., 2007). When the classical emotional Stroop paradigm is modified to include taboo words, such as swear words, those taboo words elicit even longer reaction times (Eilola et al., 2007). The modified emotional Stroop paradigm, which includes taboo words, allows us to disentangle failures to recognize social cues from failures to control one's behavior. We use taboo words as context-free and salient social cues to control. These words are easily recognized and are inappropriate in most communication contexts.

During the emotional Stroop task, the task-irrelevant information in the presented words (i.e., valence or taboo–quality of the words) interferes with the task of naming the color of the words. Self-control is necessary to counteract this interference. Thus, whether or not depleted people recognize relevant social cues to control (i.e., taboo words) would lead to two distinct outcomes: if depletion does not impede recognizing social cues, depletion should not diminish the interference effect; thus, the reactions to negative and taboo words should be delayed relative to the reactions to the neutral words. In contrast, if depletion hinders the recognition of social cues, the interference effect should be diminished; thus, the reactions to negative and taboo words should be similar to the responses to the neutral words. Thus, the modified emotional Stroop task pinpoints where along the regulation sequence the failure occurs: at the earlier stage of cue recognition or the subsequent stage of behavior control.

## Materials and methods

### Ethics statement

We conducted this study in accordance with the APA ethical standards and the German Psychological Society's (DGPs) ethical guidelines (2004, CIII). According to the DGPs' ethics commission, an institutional research board's ethical approval is only required if any funding is subject to such an ethical review. No such requirements were present for this study. Participation in the study was voluntary, no reward or incentive was granted apart from research participation time attested to students at the psychology-department of the University of Freiburg. All participants were told beforehand about the presentation of taboo words in the course of the study and gave informed consent about this as well as the usage of their provided data upon entering the study. Participants were also made aware that they could abort the study at any time without any repercussions. All data was collected and analyzed anonymously.

### Participants and design

Participants were recruited from various social media platforms (Facebook and online-forums) and student participant mailing lists. No reward was offered except for a participation confirmation for students at the authors' institute. In total, 854 participants participated in the study. We excluded extreme values of the time taken to complete the entire study (*n* = 32), time taken to complete the color Stroop task (*n* = 5) and errors made in the color Stroop task (*n* = 43) to only include participants who completed the task diligently enough to be affected by the temporary depletion of their capacity to self-regulate. We also excluded participants who used a smartphone (*n* = 136) despite being instructed not to because the reaction time tasks used a fixed layout that does not display well on narrow screens. Some participants met several exclusion criteria. The final sample included *N* = 650: 500 women (76.9%), 102 men (15.7%), and 48 of unknown gender (7.4%). The participants were randomly assigned to the depletion condition (*n* = 340) or the control condition (*n* = 310).

### Procedure

The participants were greeted and informed that they will be shown offensive words during the study. After obtaining informed consent, the participants answered demographic questions regarding their age, sex, level of education, whether they have studied or are studying psychology, and which input device they use (mouse, touch, or trackpad). Next, the participants completed the German short version of the self-control-scale (SCS-K-D; Bertrams and Dickhäuser, 2009) and six items regarding their implicit theories on willpower (Job et al., 2010). Participants in the depletion group completed a modified color Stroop task with predominantly incongruent trials. Participants in the control group completed a modified color Stroop task with predominantly congruent trials. Next, the participants were asked about their experiences and feelings regarding the task. Finally, the participants completed the emotional Stroop task and were again asked about their experiences and feelings regarding the task.

### Materials

#### Self-control scale

We included the German short version of the self-control-scale (SCS-K-D; Bertrams and Dickhäuser, 2009) as a covariate. This scale measures self-control capacity as a trait. The 13 items describe successful or unsuccessful regulation and regulation relevant behavior, such as “I'm good at resisting temptations.” In our study, the scale had an internal consistency of Cronbach's α = 0.79.

#### Implicit willpower theories (ITWP)

We included the German version of six items that capture the participants' implicit theories on “willpower” (in the sense of self-control; Job et al., 2010). These items ask the participants if they believe that their willpower can be depleted or if they believe that their willpower is unlimited. These items were included as a potential moderator of the ego depletion effect based on a study performed by Job et al. (2010), who found that the belief in unlimited willpower mitigated ego depletion. The six items are balanced, with three items implying depletable willpower and three items implying unlimited willpower. This balance among the items was incorporated to ensure that the items do not prime the participants in one direction. For example, one item asks the following: “After you have completed a difficult task, you are not able to continue with something new with the same concentration. You have to recover first.” In our sample, this scale had an internal consistency of Cronbach's α = 0.82.

#### Color stroop task

A modified color Stroop task was used to induce ego depletion. The stimuli were color words (i.e., RED, GREEN, BLUE, and YELLOW) displayed in one of these four colors. The stimuli were either incongruent (word meaning and displayed color differed) or congruent (word meaning and displayed color matched). Each word was displayed until the participants responded. Then, a fixation cross appeared, followed by the next word. The participants responded by clicking one of four buttons below the stimulus area. The buttons were labeled “red,” “green,” “blue,” or “yellow” in black text. The button order was randomized for each participant. The participants were asked to indicate the displayed color of the word, while ignoring the meaning of the word. For example, the correct answer to the word “RED” displayed in green is green. The task consisted of 64 words, with an equal distribution of colors. The participants in the depletion group completed 52 incongruent trials and 12 congruent trials. The participants in the control group completed 12 incongruent trials and 52 congruent trials.

#### Emotional stroop task

An emotional Stroop task was used as the dependent measure. The stimuli included three different word types: words with neutral valence (20), words with negative valence (20), and taboo words (19); this task was based on studies performed by Eilola et al. (2007). The words were displayed in one of four colors (i.e., red, green, blue, or yellow). The interface and procedure was identical to the color Stroop task; however, the stimuli and instructions differed. All participants were presented the full list of words throughout the task and thus completed trials with all three word types. Reaction times were aggregated separately for neutral, negative, and taboo words. Only correctly answered trials were aggregated (Ratcliff, 1993). Reaction times were aggregated using the harmonic mean, which is less sensitive to outlier reaction times than the arithmetic mean (Ratcliff, 1993; Singh and Göritz, submitted).

#### Control items after color stroop and emotional stroop

We used a set of control questions after each reaction time task to ask the participants about their experiences and feelings regarding the task. Four questions were used in the semantic differential format to ask the participants if they considered the task difficult—easy, effortful—effortless, monotonous—varied, and pleasant—unpleasant. The participants' current mood was measured using the affect scale of the Self-Assessment Manikin (SAM; Bradley and Lang, 1994), which is a pictorial assessment technique. The SAM uses five stylized manikins that differ in their facial expression from a deep frown to a bright smile.

## Results

### Preliminary analyses

To determine whether the online emotional Stroop was successful, we examined whether we replicated the pattern found in prior studies using our control condition (i.e., non-depletion). Similar to prior studies, the participants were significantly slower to indicate the color of taboo words than the color of neutral or negative words, *F*_(2, 571)_ = 14.14, *p* < 0.001, $\eta_{p}^{2}$ = 0.047. Moreover, the participants were somewhat slower to indicate the color of negative words than neutral words. However, this difference was only significant at the 10%-level (*p* = 0.094). The participants completed the emotional Stroop task diligently: Of a total of 59 trials, the median number of errors was one.

To determine whether the online color Stroop task worked as intended, we examined whether we replicated the classic Stroop interference effect (MacLeod, 1991): regardless of the depletion condition of the color Stroop task, the participants responded significantly slower during the incongruent trials than during the congruent trials, *F*_(1, 648)_ = 1549.02, *p* < 0.001, $\eta_{p}^{2}$ = 0.705, thus replicating the classic Stroop effect.

Additionally, we analyzed whether depletion affected the participants' mood by comparing the depleted and undepleted groups. The participants in the depleted group rated the color Stroop task as more difficult, *t*_(607.99)_ = 2.20, *p* = 0.028, more exhausting, *t*_(608)_ = 2.86, *p* = 0.004, and more unpleasant, *t*_(608)_ = 2.00, *p* = 0.046, than the participants in the undepleted group. However, no significant effect on mood was observed, *t*_(608)_ = 0.1, *p* = 0.922. The emotional Stroop task showed no significant differences in both reception and mood-induction between the depleted and undepleted participants. Therefore, we are confident that while the depleting task was perceived as more taxing than the non-depleting task, any ego-depletion effects observed in the emotional Stroop task are not due to mood differences between the depletion conditions.

### Main analyses

To determine whether ego depletion reduces the participants' sensitivity to negative and taboo words, we examined whether the emotional Stroop effect is moderated by ego depletion. We performed a 3 × 2 mixed-factor ANCOVA with word type (neutral vs. negative vs. taboo) as a 3-level within-subjects factor and depletion condition (depletion vs. non-depletion) as a 2-level between-subjects factor. The dependent variable was the harmonic mean of the reaction times (RT) of the correct trials in the emotional Stroop task. We included trait self-control as a covariate to account for individual differences in the ability to self-regulate, *F*_(1, 572)_ = 9.74, *p* = 0.002, $\eta_{p}^{2}$ = 0.017.

The word type alone did not affect the RT, *F*_(2, 1144)_ = 0.97, *p* = 0.381, $\eta_{p}^{2}$ = 0.002. In addition, the induction of ego-depletion alone did not affect the RT, *F*_(1, 572)_ = 2.61, *p* = 0.106, $\eta_{p}^{2}$ = 0.005. However, the interaction between depletion and word type was significant, *F*_(2, 1144)_ = 3.82, *p* = 0.022, $\eta_{p}^{2}$ = 0.007. Thus, ego depletion significantly moderated the emotional Stroop effect.

To separate the factor levels, we performed two simple effects analyses. We applied the Sidak-adjustment to correct for the alpha-error-inflation (Field, 2013).

First, we compared the difference between the depletion and non-depletion conditions separately for each word type. We assumed that a reduction in the emotional Stroop effect would be observed in the depleted participants, i.e., the depleted participants should have a lower harmonic mean RT when presented with taboo and negative words in the emotional Stroop task than the undepleted participants. Our hypothesis was confirmed as follows: In the depletion condition, no significant difference was observed among the three word types, *F*_(2, 571)_ = 1.59, *p* = 0.205, $\eta_{p}^{2}$ = 0.006. Thus, depleted participants do not react at a different speed to words of different types.

In contrast, in the non-depletion condition, the RT differs significantly among the three word types, *F*_(2, 571)_ = 14.14, *p* < 0.001, $\eta_{p}^{2}$ = 0.047. The RT to taboo words differs significantly from that to neutral words [*p* < 0.001, 95%-CI (−61.10; −22.97)] and negative words [*p* = 0.002, 95%-CI (−44.09; −7.42)]. The difference between neutral and negative words approaches significance in the expected direction, *p* = 0.094, 95%-CI [−34.46; 1.89]. Thus, the undepleted participants reacted the fastest to neutral words, slightly slower to negative words, and significantly slower to taboo words, which represents the classic emotional Stroop effect.

Second, we compared each word type separately for the depleted and undepleted conditions.

**Figure 1:**
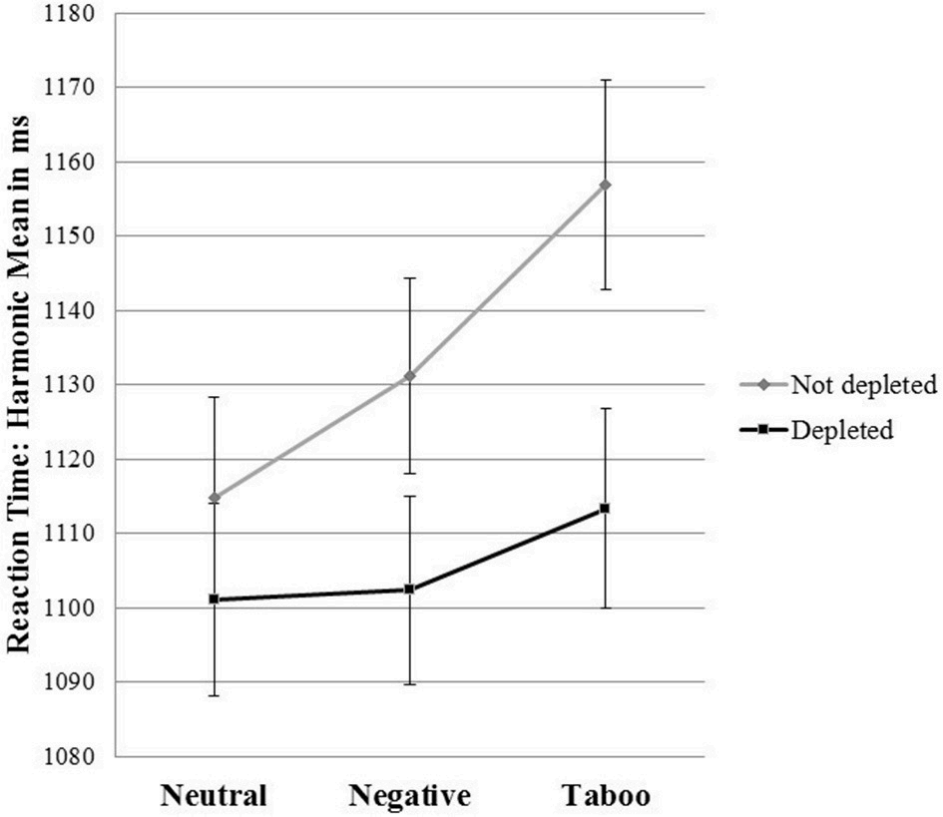
Interaction effect between word type and depletion condition.

We found no significant difference between the undepleted and depleted conditions with neutral words [(*p* = 0.458, 95%-CI −22.85; 50.60)] and negative words [*p* = 0.116, 95%-CI (−7.12; 64.76)]. However, our data show a tendency toward shorter RTs with the negative words in the depleted participants. We found a significant difference between the undepleted and depleted conditions with taboo words [*p* = 0.026, 95%-CI (5.30; 81.84)]. The depleted participants showed a significantly diminished delay to taboo words compared with the undepleted participants (Figure 1).

We also tested whether the effects were moderated by the implicit theories on willpower the participants held. To perform this analysis using a within-subjects design, we recoded the ITWP-score into three factor levels: low (≤−1 SD), medium, and high (≥+1 SD). The recoded variable was entered into the model as a between-subjects factor. The ITWP had no significant effect on the RT as a function of word type, *F*_(2, 590)_ = 0.19, *p* = 0.827, $\eta_{p}^{2}$ = 0.001. Moreover, the ITWP showed no significant interaction with the depletion condition, *F*_(2, 590)_ = 1.32, *p* = 0.267, $\eta_{p}^{2}$ = 0.004. Finally, the threefold-interaction among ITWP, depletion condition and word type in the emotional Stroop task did not reach significance, *F*_(4, 1108)_ = 0.48, *p* = 0.749, $\eta_{p}^{2}$ = 0.002. We conclude that the ITWP do not moderate the RT regardless of word type, depletion condition or their combination.

## Discussion

### Study summary

We tested whether a reduced self-control capacity makes it harder for people to recognize social cues to control. We experimentally assigned people to two versions of a color Stroop task: one version depleted the participants' self-regulation capacity, and the other version did not deplete this capacity or depleted it to a lesser degree. Then, the participants' reactions to social cues in the form of different types of words (i.e., neutral valence, negative valence, taboo words) were tested using a modified version of the emotional Stroop task.

The results support our hypotheses: Strong social cues in the form of taboo words are processed differently from weaker cues or neutral stimuli in the form of negative or neutral words. Therefore, social cues manifest in text-based communication. The depleted participants react faster and, thus, differentiated less among the textual social cues with different valences. In contrast, the undepleted participants reacted more slowly to the taboo cues than to the neutral or negative cues; thus, these participants differentiate more (appropriately) among different textual cues.

### Primary goals

Our study had two main goals: (1) to experimentally demonstrate that lapses in self-control decrease peoples' sensitivity to social cues to control and (2) deliver a proof of principle that these types of experiments are feasible in online settings using technology native to most web browsers without any plug-ins.

Using the emotional Stroop task, we isolated the first step in the self-control process, namely, cue recognition. In contrast to the classic emotional Stroop effect, the RTs to taboo words were less delayed when the participants were first depleted. Therefore, the self-control failure occurred at the earlier stage of cue recognition and not at the later stage of behavior modification. The depleted participants treated taboo words similarly to neutral words, indicating that they were insensitive to social cues to control (i.e., the inappropriateness of taboo words).

This finding illustrates the importance of distinguishing between the stages of the self-control process: All three types of self-control failure [(1) not intending to communicate appropriately; (2) intending to communicate appropriately, realizing behavior should be modified, but being unable to do so; and (3) intending to communicate appropriately, able to modify the behavior, but failing to realize the necessity] lead to inappropriate behavior. However, a failure to recognize relevant cues (failure of type 3) indicates that the failure is not consciously represented by the participants because the control process was never activated. In our experiment, taboo words did not elicit a different reaction than harmless words in the depleted participants.

Failing to recognize cues can prevent the strengthening that occurs during the later stages of self-control from having an effect. Thus, among people who are less able or unable to appropriately recognize cues to control, interventions that reaffirm norms regarding appropriate communication are less effective. If people do not realize that a communication situation warrants self-control, their motivation to communicate appropriately is irrelevant. Similarly, if impulse control is never (fully) activated, interventions that help people overcome the impulse to communicate inappropriately are ineffective.

Regarding the feasibility of a Stroop-based experiment in an online setting, our results are promising. The participants reported little to no trouble completing the tasks. Furthermore, the modified emotional Stroop task was successful: taboo words led to significant RT increases in the non-depletion condition. Web-based RT paradigms may help unravel more of the mechanisms of online disinhibition in future studies. These paradigms allow for explorations of the aspects of online behavior that researchers cannot measure using self-report measures. Furthermore, collecting data in online settings is faster, cheaper and can reach a wider demographic than most laboratory studies (Göritz and Schumacher, 2000). Furthermore, online data collection is less affected by social desirability (Joinson, 1999), and participants are recruited “in the field.” Regarding OD, online studies have the special benefit of being conducted in the very setting in which the relevant behavior occurs.

### Limitations and future research

Based on our study, there are several avenues for further studies. In this study, the depleted participants treated taboo words and neutral words similarly. Therefore, taboo words drew the same attention as neutral words from depleted participants. In addition to exploring RTs to cues, impaired cue recognition could be investigated by identifying the cues that are recalled more easily after the task. Recalling taboo words (which are more striking than negative words and even more striking than neutral words) should be more successful than recalling neutral words. If the failure of cue recognition found in this study extends to cue recall, depleted people should be able to recall neutral and taboo words equally well.

Another approach for future studies is to test whether depletion increases the actual usage of taboo words. In this study, we chose the emotional Stroop task to differentiate between the failure to recognize social cues and the failure to inhibit impulses despite attempting to. The failure to react to taboo words likely implies that the participants would have a harder time avoiding using taboo words. This assumption posits that the same monitoring process governs the recognition of inappropriate words regardless of whether the words are read or heard or whether we consider using them. Future studies could test whether depleted participants are more likely to use taboo words than undepleted participants. This study could be performed using actual text production or by allowing participants to select words under time constraints.

## Conclusion

We believe that OD studies could profit from the self-control perspective of disinhibited online communication in two ways: (1) Identifying the hotspots of OD by exploring where on the Internet self-control capacity is lacking and (2) examining the specific contexts in which OD occurs and determining the types of self-control failures, which may provide deeper insights into the causes of OD and potential interventions.

One approach to gauge the level of available self-control capacity in a particular online context is to explore self-control capacity from a trait perspective. Different online communities may attract people with different self-control capacities, which might help in identifying communities that are at risk of toxic communication patterns. Toxic communication could occur in communities with members from demographic groups with a lower average self-regulation capacity, such as younger or less educated people (de Ridder et al., 2012). Toxic communication might also occur in communities with members from demographic groups that have an average self-control capacity but have to rely more heavily on self-control due to stronger adverse impulses, such as men (de Ridder et al., 2012). For example, communities formed around competitive online gaming are notorious for their toxic communication (Ballard and Welch, 2017).

The users' available self-control capacity may also depend on the (physical) Internet usage contexts (Döring, 2003). The following questions must be considered: What devices are used? What are the circumstances? What is the location? How is the Internet used? Using the Internet on a desktop PC in the office for a work-related goal might be more likely to facilitate self-control than using a smartphone for informal communication on the subway. We could expect the context to impair self-control in online communications (1) if the context holds parallel demands for self-control (e.g., distracting environments), (2) if the context deemphasizes self-monitoring or self-control (e.g., usage at home), or (3) if the context is preceded by strong self-control exertion (e.g., after a long workday).

Finally, the users' available self-control capacity may depend on the properties of the relevant communication platform. The design of an app or website likely has an impact on the average available self-control capacity of its users. The design includes aspects of the interface (e.g., ease of use), visual appearance (e.g., readability), and community features of the platform (e.g., reputation systems). These aspects can be examined to determine whether they place extraneous self-control demands on users (e.g., a confusing interface). Furthermore, these aspects may offer means to facilitate user self-control. In fact, certain systems, such as reputation systems (e.g., Reddit's karma points), and feedback systems (e.g., Liking on Facebook) are self-control interventions because they emphasize self-monitoring and facilitate social sanctions.

In addition to identifying when and where lapses of self-control are more likely, exploring specific instances of OD from a self-control perspective is warranted. OD in each context raises the question of which types of self-control failures are primarily responsible. Answering this question narrows the search for possible causes and solutions.

Regarding the first type of self-control failure in which users in an online community do not intend to communicate appropriately, selection effects or dysfunctional community norms could be further investigated. For example, the community may attract many people with anti-social tendencies or pronounced traits of the dark tetrad due to its topics or the channels of recruitment. The prevalent patterns of toxic communication in a community may perpetuate themselves by self-selection (i.e., members who value politeness leave) and socialization (i.e., new members assume that OD is appropriate). This situation requires specific solutions, such as changing the member composition, changing or clarifying the desirable norms, or introducing consequences. Changing the member composition may entail recruiting efforts to draw in new members with desirable social interaction styles or banning particularly toxic members. Changing and clarifying the norms might involve making the norms more explicit (e.g., in a netiquette; i.e., a set of rules for good conduct online) or having moderators reiterate the rules and standards repeatedly in different posts or threads. Finally, introducing consequences might include banning members. However, less drastic measures may be realized using reputation and rating systems. These systems can be used to either introduce negative consequences for OD, positive consequences for markedly constructive communication, or both. The online discussion platform Reddit's karma system is an example.

The other two types of self-control failures can only occur if users are willing to comply with the standards of constructive communication. In this case, instances of OD indicate that the users were either unable to inhibit inappropriate communication or failed to realize that the communication was inappropriate. The question of whether users who communicated inappropriately attempted to avoid OD disentangles the two forms. If self-control fails at the later stage of behavior modification, the inappropriate nature of the intended communication has been realized, but users may find themselves unable to stop it. If self-control failed at the earlier stage of recognizing social cues, the norm violation is not consciously represented, and hence, no attempt is made to modify one's behavior. From an epidemiological perspective, a high prevalence of cue recognition failures should result in a large discrepancy between objective measures of OD (counting OD comments) and subjective measures of OD (asking users about the extent of perceived OD or their own OD).

Both types of self-control failures in which people are willing to communicate appropriately share certain causes and, hence, solutions. The previously discussed factors that reduce the available self-control capacity of users in a community can lead to both types of failures. A reduced self-control capacity lowers the likelihood that users recognize when control is necessary. However, some users still recognize when control is necessary but because of the lower self-control capacity, they may fail at the stage of inhibiting the inappropriate communication behavior. Consequently, the previously discussed approaches to increase the available self-control capacity in a community are applicable.

However, there are also factors and solutions that are specific to either of the two types of self-control failures in which people are willing to avoid OD. Failures to inhibit inappropriate communication may become more likely if aspects of the community increase impulse strength (i.e., the urge to communicate inappropriately). The likelihood of the successful inhibition of a behavioral impulse depends on both the self-control capacity and the strength of the impulse (de Ridder et al., 2012). Therefore, self-control failure becomes more likely if the impulse becomes stronger even if the self-control capacity is the same. This situation could occur in communities that discuss controversial topics and draw members from different sides of an ideological divide. Examples include communities that are dedicated to political discussions or the reader comment section of an online newspaper. The prevalence of targeted, intentional insults in these discussions (e.g., Alonzo and Aiken, 2004) demonstrates that failures of inhibition occur in addition to failures of cue recognition. After all, an intentional, targeted insult tailored to a communication partner requires an individual to select an insult based on the inappropriate and hurtful nature of the message. If the conditions in a community lead to stronger impulses toward inappropriate communication, the solutions should focus on proactively diffusing conflicts. Timely moderation and deletion of inappropriate posts may prevent other users from becoming incited and reacting aggressively in response to such posts. Similarly, rules against arguing ad hominem could be reiterated and enforced consistently.

In contrast, failures to recognize social cues may be more likely if the social norms and roles in a community are ambiguous. Social cues are easier to identify in a community with homogenous members and clear community goals and rules. In addition to strengthening the available self-control, solutions for failures to recognize cues could involve supporting self-monitoring. This solution could be achieved by peer feedback (via rating systems), moderator feedback, or automated solutions (e.g., via automatic recognition of inappropriate words). Solving the problem of failures to recognize cues involves the additional challenge of users who do not realize that they behave inappropriately. Before solutions are implemented, it may be necessary to first convince users of the problem and their role in it.

In summary, we believe that the self-control capacity perspective and distinguishing different types of self-control failures offer a new perspective of OD. This perspective should inspire future studies and may lead to more carefully targeted practical solutions to address OD in online communities.

## Author contributions

BV: Finalizing conception and design of the work; acquisition, analysis and interpretation of data; drafting, revising and final approval; Agrees to be accountable for all aspects of the work. RS: Original conception and design of the work; acquisition of data; drafting, revising, and final approval; Agrees to be accountable for all aspects of the work. AG: Conception of the work; revising and final approval; Agrees to be accountable for all aspects of the work.

### Conflict of interest statement

The authors declare that the research was conducted in the absence of any commercial or financial relationships that could be construed as a potential conflict of interest.
